# The Complete Chloroplast Genome of Tornillo (*Cedrelinga cateniformis* Ducke 1922, Fabaceae)

**DOI:** 10.1002/ece3.71355

**Published:** 2025-04-23

**Authors:** Nora Scarcelli, Cédric Mariac, Marie Couderc, Diana Castro Ruiz, Guillain Estivals, Carlos Alberto Custodio Angulo Chavez, Hector Acho Vasquez, Jhon Gregory Alvarado Reategui, Tony Vizcarra Bentos, Carmen Garcia‐Davila

**Affiliations:** ^1^ DIADE, Univ Montpellier, Cirad, IRD Montpellier France; ^2^ Instituto de Investigaciones de la Amazonía Peruana (IIAP), Laboratorio de Biología y Genética Molecular (LBGM) Iquitos Peru

**Keywords:** *Cedrelinga cateniformis*, chloroplast genome, Fabaceae, tornillo

## Abstract

Tornillo (*Cedrelinga cateniformis* Ducke 1922) is a tropical tree of the Fabaceae family. It is commonly used in the lumber factory and is an interesting substitute to overexploited tropical timber species. We sequenced and assembled the first whole chloroplast genome of Tornillo, using Oxford Nanopore technology. The Tornillo's chloroplast is a circular molecule of 176,700 bp with 138 genes and a classic quadripartite structure. The Inverted Repeats present a huge expansion, combined to a strong reduction of the Short Single Copy. Similar results were previously observed in other species of the tribe Ingeae. A maximum likelihood phylogenetic tree reveals a clear distinction of the Ingeae tribe from other tribes (Acacieae and Mimoseae) of the Fabaceae family.

## Graphical abstract

Tornillo (Cedrelinga cateniformis) is a tropical tree from the Fabaceae family, valued as a substitute for overexploited timber species. This study presents the first complete chloroplast genome sequencing of Tornillo, revealing a 176,700 bp structure with 138 genes and confirming its phylogenetic placement within the Ingeae tribe.

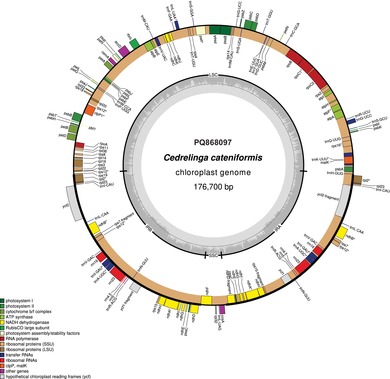

## 1 Introduction

**FIGURE 1:**
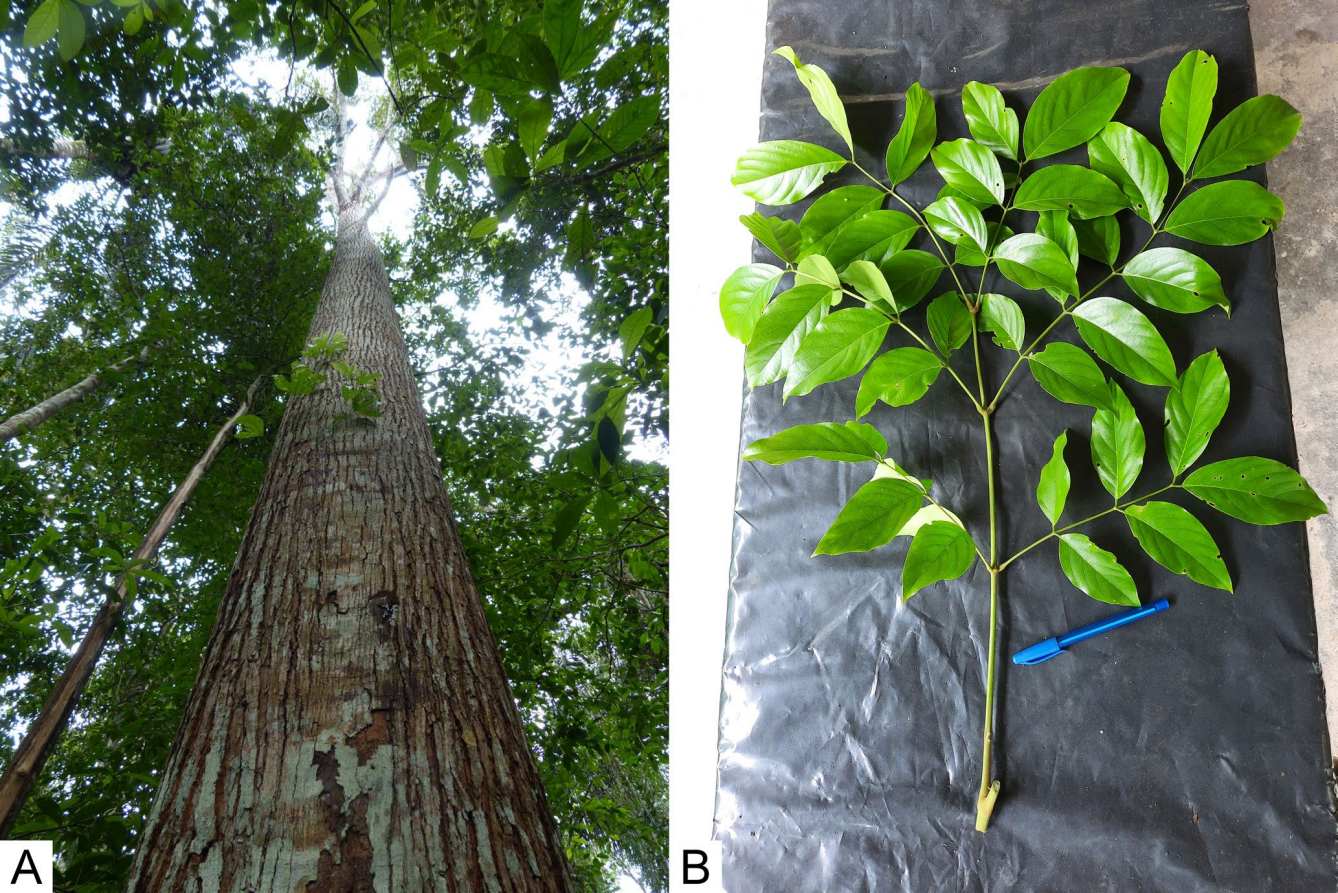
Tornillo tree (A) and details of young leaves (B). Photos taken by T. Vizcarra Bentos in the IIAP research centre of Jenaro Herrera.

*Cedrelinga cateniformis* Ducke 1922 is a tropical tree species of the family Fabaceae (Figure 1) commonly known as ‘Tornillo’ in Peru. Tornillo has a wide ecological distribution in humid tropical, subtropical and dry tropical environments. It usually grows in environments with annual precipitations ranging from 2500 to 3800 mm and average temperatures from 23°C to 38°C. It is reported in the Amazon regions of Ecuador, Peru, Colombia and Brazil, from 120 to 800 m above sea level (Cruz et al. 2020). Due to its rapid growth in natural and managed environments, Tornillo is considered a secondary succession species (Baluarte‐Vásquez and Alvarez‐Gonzales 2015; Guariguata et al. 2017).

According to FAO (2018), Tornillo is one of the main species used as sawn wood from tropical forests. Its timber potential for various uses in the furniture and construction industry (Haag et al. 2020), as well as its suitability for restoring degraded areas (Rojas Briceño et al. 2020), places it as an economic alternative for cultivation in agroforestry or polyculture systems. Tornillo's wood is easy to work with, providing a good finish, which explains its use in carpentry (Gonçalez and Gonçalves 2001). As it is now considered a substitute for overexploited tropical species (Haag et al. 2020), it is urgent to draw a strategy for its exploitation and conservation. Yet, there are very few studies on its genetic diversity and few genetic resources are available (Cruz et al. 2020). In an attempt to increase these genetic resources, we present here the first complete chloroplast genome of *Cedrelinga cateniformis*.

## 2 Materials and Methods

We collected fresh leaves from a single Tornillo tree in the research centre of Jenaro Herrera, IIAP, Peru, at the coordinates −4°53′59.4024 N/−73°38′55.7016E (WGS84). The voucher MERP930 of the plant is available in the Herbario Herrerense (Data S1. Herbarium HH, headquarters Iquitos, contact Dr. Dennis del Castillo Torres, ddelcastillo@iiap.gob.pe).

We extracted DNA of high molecular weight following Scarcelli et al. (2024). We then constructed a single library using the ligation sequencing kit DNA SQL‐LSK110 (Oxford Nanopore Technology), following the constructor's recommendations. We sequenced the library on an R9.4 flowcell, using a MinION Mk1B. The base calling was done using the high accuracy model of Minknow 7.1.4 + d7df870c0 with the configuration file dna_r9.4.1_450bps_sup.cfg. Only high‐quality reads (*Q*‐score > 10) were kept for the following steps.

We used the pipeline ptGAUL 1.0.5 (Zhou et al. 2023) to generate the whole chloroplast genome assembly. This pipeline first discarded non‐chloroplast reads, based on alignment on a chloroplast reference using Minimap2 (Li 2018). We used *Albizia julibrissin* (NC_058305.1) and *Senegalia senegal* (NC_045513.1) for reference because, at that time, these two species were the closest species to *C. cateniformis* with a whole chloroplast available. The pipeline then discarded short reads (< 1000 bp) and reduced the coverage to 50X (Data S2). Finally, the pipeline used Flye (Kolmogorov et al. 2019) to generate the whole genome and Racon (Vaser et al. 2017) to polish it. We annotated the whole chloroplast genome using GeSeq (Tillich et al. 2017), with *Albizia odoratissima* (NC_034987.1) as reference and default parameters. Finally, we manually checked the annotations using Geneious Prime 2023.2.1. We observed 38 missing bases in ORF (0.021% of total sequence length), all in homopolymer regions. This type of sequencing error is common for ONT sequencing and well documented (Delahaye and Nicolas 2021). We therefore included 38 N to correct these missing bases.

We generated a phylogenetic analysis with 11 species of the Fabaceae family (Table 1). Species were chosen to span all the tribes of the Mimosoideae sub‐family, except Mimozygantheae because no complete chloroplast had been published at that time. Within each tribe, species were chosen at random, except for *Albizia julibrissin* and *Senegalia senegal*, which were already chosen for the chloroplast assembly. Briefly, we aligned whole chloroplast genomes with MAFFT 7.505 (Katoh and Standley 2013). Then we performed a Maximum Likelihood phylogenetic analysis with RAxML 8.2.12 (Stamatakis 2014), using the GTRGAMMA substitution model, *Citrus limon* (NC_034690.1) as outgroup and 100 bootstraps.

**TABLE 1:** List of species used to perform the phylogeny.

Species	Accession	Tribe
*Cedrelinga cateniformis*	PQ868097	Ingeae
*Albizia julibrissin*	NC_058305.1	Ingeae
*Samanea saman*	NC_034992.1	Ingeae
*Pararchidendron pruinosum*	NC_035348.1	Ingeae
*Sphinga acatlensis*	NC_047398.1	Ingeae
*Mimosa pudica*	NC_042921.1	Mimoseae
*Leucaena trichandra*	NC_028733.1	Mimoseae
*Prosopis cineraria*	NC_049133.1	Mimoseae
*Senegalia senegal*	NC_045513.1	Acacieae
*Vachellia nilotica*	NC_045514.1	Acacieae
*Citrus limon*	NC_034690.1	Rutaceae

All code lines used to perform analyses are available in Data S3.

## 3 Results and Discussion

**FIGURE 2:**
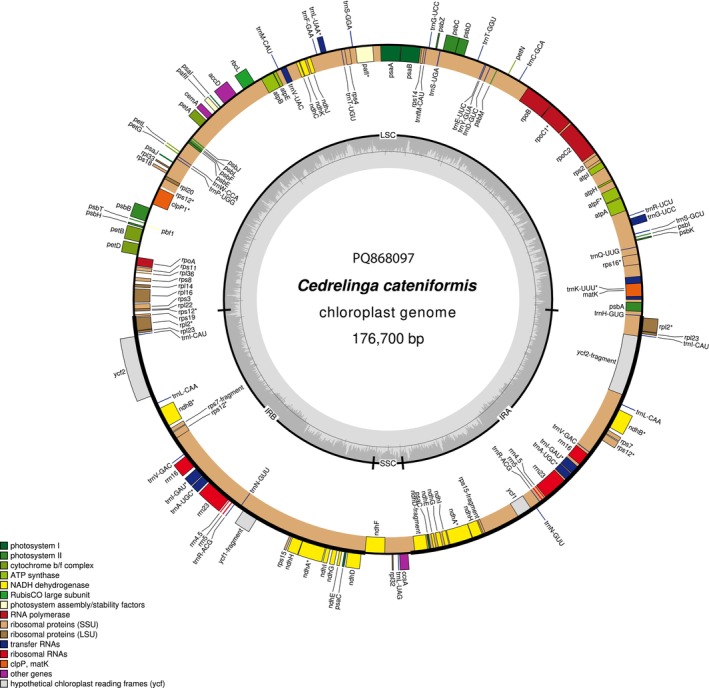
Genome map of *Cedrelinga cateniformis* chloroplast PQ868097, drawn using ORGDRAW (Greiner et al. 2019). Inner genes are translated in a clockwise direction; outer genes are translated counterclockwise. The inner circle represents the GC content. Genes marked with an asterisk contain at least one intron.

The complete chloroplast genome of Tornillo (*Cedrelinga cateniformis*) was assembled as a circular molecule of 176,700 bp with a classic quadripartite structure (Figure 2). The Large Single Copy (LSC) spanned 92,034 bp and contained 32.9% GC. The Short Single Copy (SSC) was only 5010 bp with the lowest GC content (28.7%) while the two inverted Repeats (IRa and IRb) spanned 39,828 bp each, with the highest GC content (38.5%).

Gene annotation recovered 112 unique genes, comprising 78 protein coding genes, 30 tRNA and four rRNA. 26 genes were duplicated in the IRs: *ndh*A, *ndh*B, *ndh*D, *ndh*E, *ndh*G, *ndh*H, *ndh*I, *psa*C, *rpl*2, *rpl*23, *rps*7, *rps*12, *rps*15, *ycf*1, *ycf*2, *trn*A‐UGC, *trn*I‐CAU, *trn*I‐GAU, *trn*L‐CAA, *trn*N‐GUU, *trn*R‐ACG, *trn*V‐GAC, *rrn*16, *rrn*23, *rrn*4.5 and *rrn*5. The trans‐splicing gene *rps*12 showed a complex but classical structure (Data S4): exon 1 was located in the LSC, while exons 2 and 3 were duplicated and inverted in the IRs. This structure produced two transcripts, one with exon 1 and 2/3 from IRa and the other with exon 1 and 2/3 from IRb.

The chloroplast structure of *Cedrelinga cateniformis* was similar to that of other species of the tribe Ingeae. However, compared to the other species of the Fabaceae family, all the species of the tribe Ingeae analysed here (*Cedrelinga cateniformis*, *Albizia julibrissin* , *Samanea saman* , *Pararchidendron pruinosum* and *Sphinga acatlensis*) present a huge expansion of the IRs, combined with a strong reduction of the SSC (Data S5): genes *rps*15, *ndh*H, *ndh*A, *ndh*I, *ndh*G, *ndh*E, *psa*C and *ndh*D, located in the SSC in the Fabaceae family, are duplicated and located in the IRs in the tribe Ingeae. This phenomenon was previously reported for seven species of the tribe Ingeae: *Pararchidendron pruinosum*, *Samanea saman* , *Acacia dealbata* , *Albizia odoratissima* , *Archidendron lucyi*, *Faidherbia albida* , *Inga leiocalycina* and *Pithecellobium flexicaule* (Wang et al. 2017). According to Wang et al. (2017), this expansion could be linked to an extremely AT‐rich region 100 bp upstream of the IR/SSC junction. We observed a similar pattern for the five Ingeae species analysed here, with a mean of 93% of AT in the 100 bp upstream of the IR/SSC junction.

**FIGURE 3:**
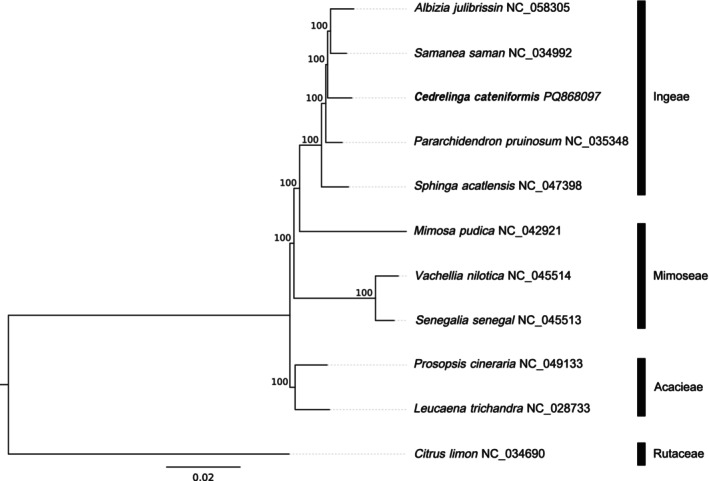
Phylogenetic tree generated by RAxML with bootstrap values indicated on nodes.

A Maximum Likelihood phylogenetic tree was performed with five species of the tribe Ingeae and six species of another tribe of the Fabaceae family (Table 1; Figure 3). The phylogenetic tree obtained was well supported and fully resolved. As expected, the five Ingeae species clustered together with high confidence and are well separated from species of other tribes (Acacieae and Mimoseae). A similar result was previously observed using a combination of plastid and nuclear markers (Ferm et al. 2021).

## 4 Conclusion

Despite its importance in the wood industry, little genetic resource is available for the species *Cedrelinga cateniformis*. Here we used Oxford Nanopore long‐read technology to reconstruct the first complete chloroplast sequence of the *Cedrelinga* genus. This will help monitor the species diversity and help set conservation and use plans.

## Author Contributions

**Nora Scarcelli:** conceptualization (equal), formal analysis (equal), investigation (equal), methodology (equal), writing – original draft (lead), writing – review and editing (lead). **Cédric Mariac:** conceptualization (equal), investigation (equal), methodology (equal), validation (equal), writing – review and editing (equal). **Marie Couderc:** conceptualization (equal), investigation (supporting), methodology (equal), writing – review and editing (supporting). **Diana Castro Ruiz:** investigation (equal), writing – review and editing (supporting). **Guillain Estivals:** investigation (equal), writing – review and editing (supporting). **Carlos Alberto Custodio Angulo Chavez:** investigation (equal), writing – review and editing (supporting). **Hector Acho Vasquez:** investigation (equal), writing – review and editing (supporting). **Jhon Gregory Alvarado Reategui:** methodology (equal), writing – review and editing (supporting). **Tony Vizcarra Bentos:** methodology (equal), writing – review and editing (supporting). **Carmen Garcia‐Davila:** investigation (supporting), writing – review and editing (supporting).

## Conflicts of Interest

The authors declare no conflicts of interest.

## Supporting information


**Data S1.** Voucher MERP930 taken from the Tornillo tree sequenced. The tree is located in the IIAP research centre of Jenaro Herrera (WGS84 −4°53′59.4024 N/−73°38′55.7016E), Peru. The voucher is available in the Herbario Herrerense (HH, headquarters Iquitos).

**Data S2.** Coverage graph and GC contents of the reads mapped to the reference *Albizia julibrissin* (NC_058305.1). The bam file was retrieved after running Minimap2 using ptGAUL, before filtering on reads length. Graph was drawn using Qualimap (Konstantin Okonechnikov, Ana Conesa and Fernando García‐Alcalde. 2015. Qualimap 2: advanced multi‐sample quality control for high‐throughput sequencing data. Bioinformatics).

**Data S3.** Code lines used to analyse the data.

**Data S4.** Structure of the rps12 gene.

**Data S5.** Comparison of the structure of the IR and the SSC. SSC is represented in blue and IRs in orange. Grey means that there was no annotation of the IR/SSC in the original GenBank file. Clockwise genes are on top, anticlockwise on bottom.

## Data Availability

The genome sequence data that support the findings of this study are openly available in GenBank of NCBI under the accession no. PQ868097. The associated BioProject, SRA and Bio‐Sample numbers are PRJNA1208262, SRR32023740 and SAMN46171921 respectively.
