# Supplementary figures and images for: The Complete Chloroplast Genome of Tornillo (Cedrelinga cateniformis Ducke 1922, Fabaceae)
Source: Ecol Evol. 2025 Apr 23;15(4):e71355. doi: 10.1002/ece3.71355 (PMC12018703; doi:10.1002/ece3.71355)

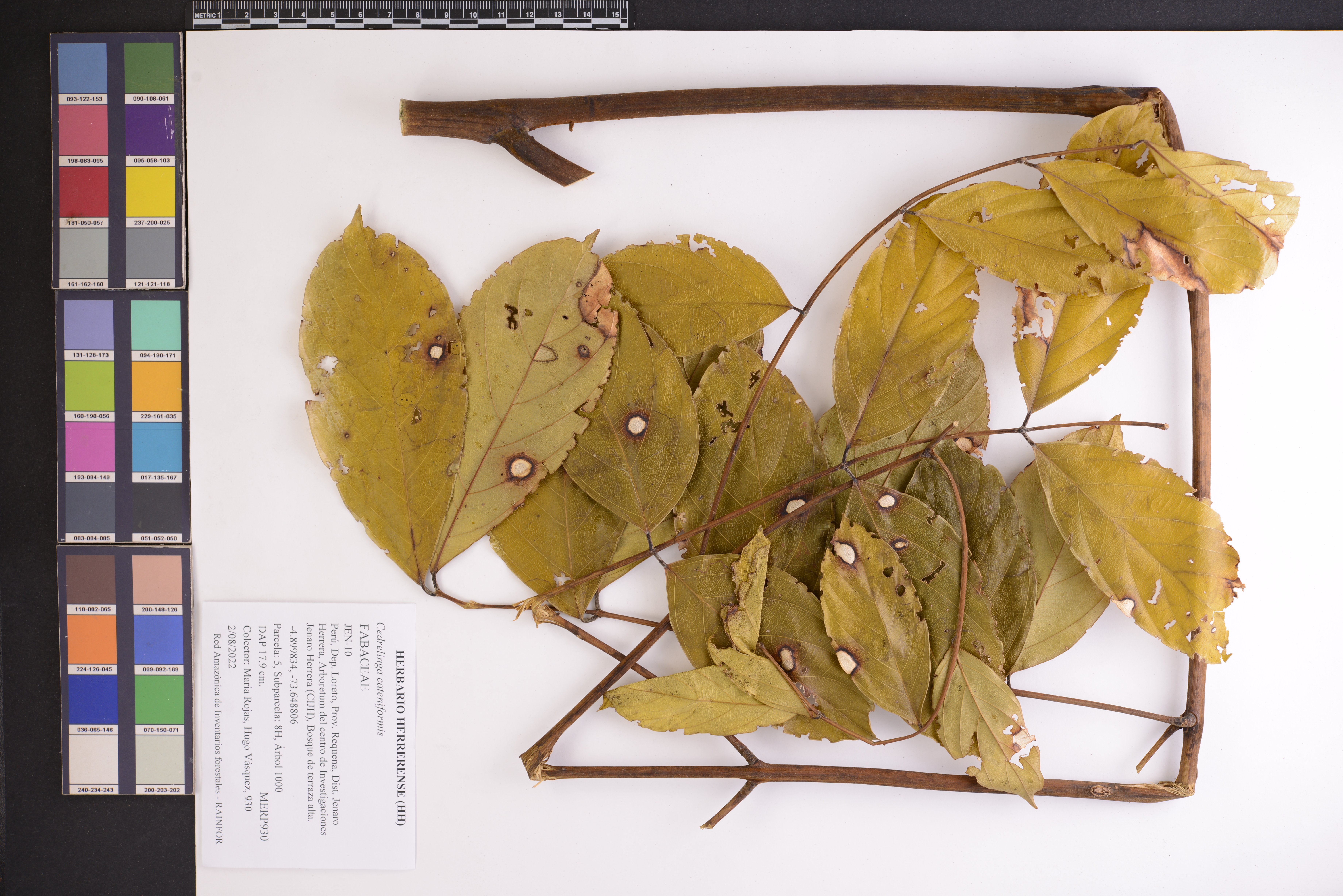

Supplement: Supplementary file 1 — Data S1. Voucher MERP930 taken from the Tornillo tree sequenced. The tree is located in the IIAP research centre of Jenaro Herrera (WGS84 −4°53′59.4024 N/−73°38′55.7016E), Peru. The voucher is available in the Herbario Herrerense (HH, headquarters Iquitos). [file ECE3-15-e71355-s004.jpg]

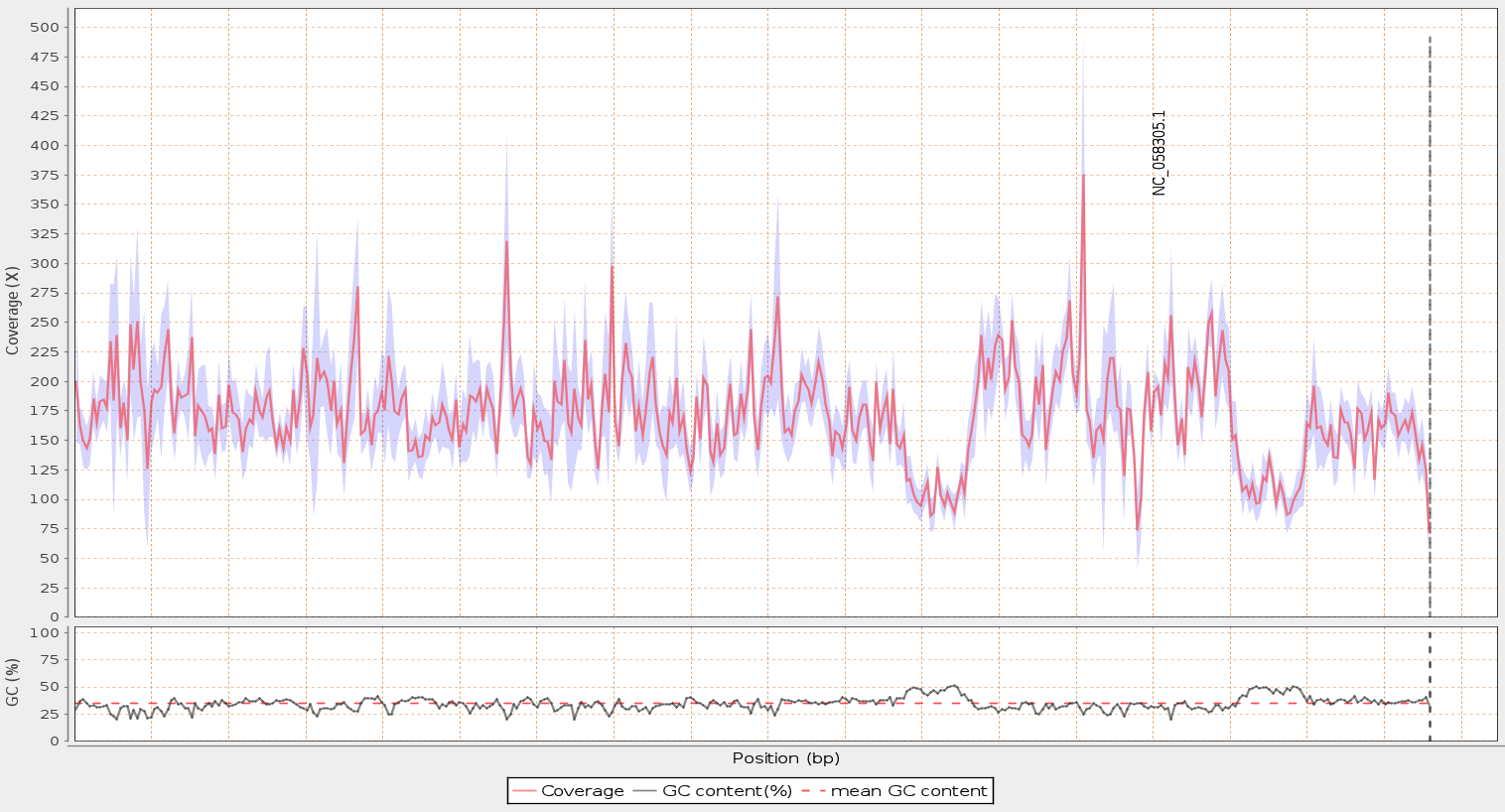

Supplement: Supplementary file 2 — Data S2. Coverage graph and GC contents of the reads mapped to the reference Albizia julibrissin (NC_058305.1). The bam file was retrieved after running Minimap2 using ptGAUL, before filtering on reads length. Graph was drawn using Qualimap (Konstantin Okonechnikov, Ana Conesa and Fernando García‐Alcalde. 2015. Qualimap 2: advanced multi‐sample quality control for high‐throughput sequencing data. Bioinformatics). [file ECE3-15-e71355-s001.png]

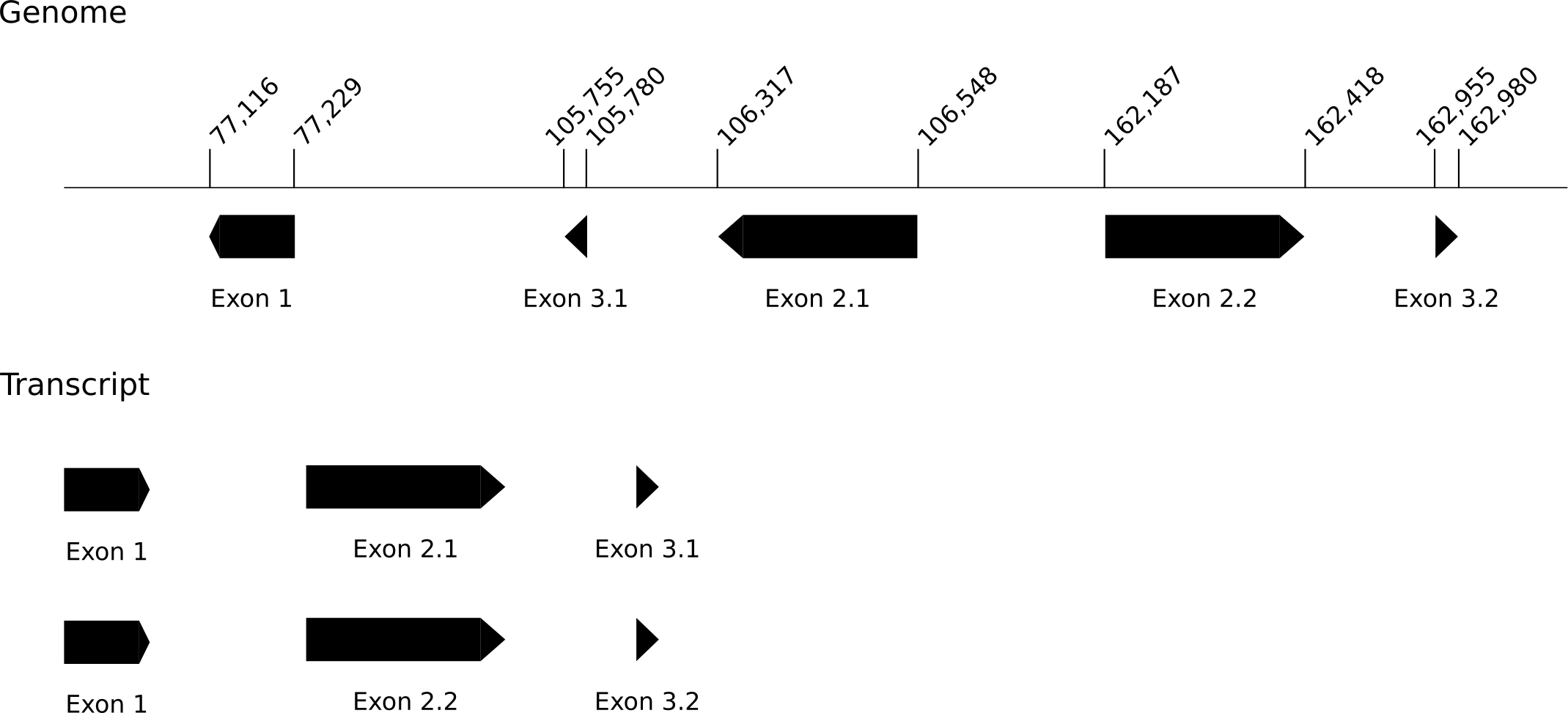

Supplement: Supplementary file 4 — Data S4. Structure of the rps12 gene. [file ECE3-15-e71355-s003.png]

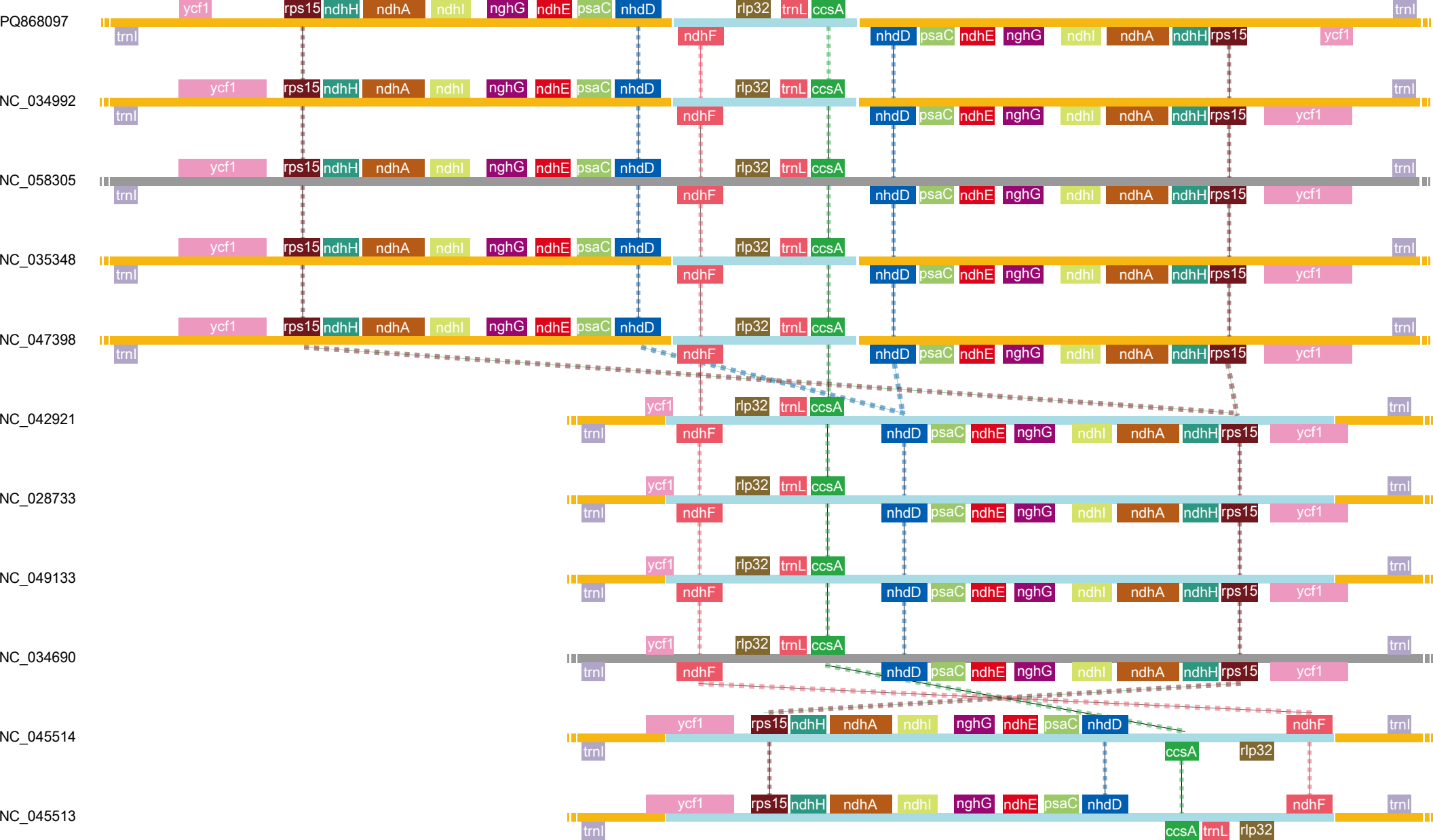

Supplement: Supplementary file 5 — Data S5. Comparison of the structure of the IR and the SSC. SSC is represented in blue and IRs in orange. Grey means that there was no annotation of the IR/SSC in the original GenBank file. Clockwise genes are on top, anticlockwise on bottom. [file ECE3-15-e71355-s005.pdf]
